# Arc 3′ UTR Splicing Leads to Dual and Antagonistic Effects in Fine-Tuning Arc Expression Upon BDNF Signaling

**DOI:** 10.3389/fnmol.2018.00145

**Published:** 2018-04-27

**Authors:** Chiara Paolantoni, Simona Ricciardi, Veronica De Paolis, Chinenye Okenwa, Caterina Catalanotto, Maria T. Ciotti, Antonino Cattaneo, Carlo Cogoni, Corinna Giorgi

**Affiliations:** ^1^European Brain Research Institute Rita Levi-Montalcini Rome, Rome, Italy; ^2^Department of Biology and Biotechnology, Sapienza University of Rome, Rome, Italy; ^3^Department of Experimental Medicine and Surgery, University of Rome Tor Vergata, Rome, Italy; ^4^Department of Cellular Biotechnologies and Hematology, Sapienza University of Rome, Rome, Italy; ^5^Institute of Cell Biology and Neurobiology, National Research Council, Rome, Italy; ^6^Bio@SNS Laboratory, Scuola Normale Superiore, Pisa, Italy

**Keywords:** Arc, EJC, splicing, BDNF, plasticity, post-transcriptional regulation, nonsense mediated mRNA decay, 3′ UTR

## Abstract

Activity-regulated cytoskeletal associated protein (Arc) is an immediate-early gene critically involved in synaptic plasticity and memory consolidation. Arc mRNA is rapidly induced by synaptic activation and a portion is locally translated in dendrites where it modulates synaptic strength. Being an activity-dependent effector of homeostatic balance, regulation of Arc is uniquely tuned to result in short-lived bursts of expression. *Cis*-Acting elements that control its transitory expression post-transcriptionally reside primarily in Arc mRNA 3′ UTR. These include two conserved introns which distinctively modulate Arc mRNA stability by targeting it for destruction via the nonsense mediated decay pathway. Here, we further investigated how splicing of the Arc mRNA 3′ UTR region contributes to modulate Arc expression in cultured neurons. Unexpectedly, upon induction with brain derived neurotrophic factor, translational efficiency of a luciferase reporter construct harboring Arc 3′ UTR is significantly upregulated and this effect is dependent on splicing of Arc introns. We find that, eIF2α dephosphorylation, mTOR, ERK, PKC, and PKA activity are key to this process. Additionally, CREB-dependent transcription is required to couple Arc 3′ UTR-splicing to its translational upregulation, suggesting the involvement of *de novo* transcribed *trans-*acting factors. Overall, splicing of Arc 3′ UTR exerts a dual and unique effect in fine-tuning Arc expression upon synaptic signaling: while inducing mRNA decay to limit the time window of Arc expression, it also elicits translation of the decaying mRNA. This antagonistic effect likely contributes to the achievement of a confined yet efficient burst of Arc protein expression, facilitating its role as an effector of synapse-specific plasticity.

## Introduction

*De novo* transcription and translation of the IEG Arc is triggered by neuronal activity and results in profound alterations of synaptic properties underlying learning and memory processes (Plath et al., 2006; Shepherd and Bear, 2011; Nikolaienko et al., 2017). Arc is a central player in synaptic homeostasis, plasticity and memory consolidation, operating at a synapse-specific level by regulating the actin cytoskeleton and the abundance of surface AMPARs in response to synaptic activity (Messaoudi et al., 2007; Bramham et al., 2010; Korb and Finkbeiner, 2011; Shepherd and Bear, 2011; Nikolaienko et al., 2017; Okuno et al., 2017). As an IEG, transcription of Arc mRNA is rapidly induced by patterns of synaptic activity that elicit LTP (Steward et al., 1998; Rao et al., 2006) and a portion is transported and locally translated in dendrites (Steward and Worley, 2001; Dynes and Steward, 2012; Farris et al., 2014; Na et al., 2016; Wang et al., 2016). The protein rapidly but transiently accumulates at inactive synapses of activated dendrites via a mechanism of “inverse synaptic tagging” thereby eliciting AMPARs endocytosis and preventing undesired strengthening of un-stimulated synapses (Chowdhury et al., 2006; Okuno et al., 2012, 2017; Zhang et al., 2015). Further, Arc plays a critical role in mGluR-dependent-LTD, however, in this case its transcriptional activation is delayed in comparison to LTP-inducing stimuli while its translation is rapidly induced (Shepherd and Bear, 2011). Recent groundbreaking studies have revealed that segments of Arc protein resemble retroviral Gag domains and mediate Arc assembly into virus-like capsids. These vesicles can capture RNA transcripts, including Arc’s own mRNA, and transfer them to post-synaptic cells. Thus, Arc is also involved in intercellular signaling and trafficking of mRNAs (Zhang et al., 2015; Ashley et al., 2018; Pastuzyn et al., 2018).

Key to the modulation of synaptic strength and balance, Arc gene expression is uniquely regulated to result in a highly transient and localized surge in Arc mRNA and protein levels upon synaptic activation. Alterations of its highly transitory expression profile profoundly affect synaptic homeostasis and memory consolidation (Plath et al., 2006; Rial Verde et al., 2006; Korb and Finkbeiner, 2011) and have been linked to neurological disorders including Schizophrenia, Fragile X syndrome, Angelman syndrome, and Alzheimer’s disease (Wu et al., 2011; Kerrigan and Randall, 2013; Purcell et al., 2014; Chuang et al., 2016; Manago et al., 2016; Pastuzyn and Shepherd, 2017). A recent study in fear memory formation, revealed that Arc expression can occur in two waves; an initial transient expression following the stimulus and a second increase in Arc levels after 12 h, which is dependent on BDNF and essential for memory consolidation (Nakayama et al., 2015). An analogous delayed translational increase in Arc levels was described during sleep-dependent consolidation of cortical plasticity (Seibt et al., 2012). Thus, Arc transient induction may occur in biphasic bursts of expression during memory consolidation, although the mechanism eliciting this delayed reactivation is still unclear.

Arc fast expression kinetic results from a complex balance of stimulatory and inhibitory mechanisms governing Arc synthesis and decay. Arc transcription is rapidly induced by BDNF and by Ca^2+^ influx upon NMDARs activation (Steward and Worley, 2001; El-Sayed et al., 2011). These signals trigger multiple cascades including intracellular PKA and ERK/MAPK signaling as well as binding of the activity-dependent transcription factors, CREB, MEF2, and SRF to Arc promoter upstream elements (Waltereit et al., 2001; Kawashima et al., 2009). Transcription initiation occurs upon release of poised RNA Polimerase II on Arc’s proximal promoter, facilitated by enhancer-derived expression of small non-coding RNAs (Saha et al., 2011; Schaukowitch et al., 2014). Activity-dependent transcriptional activation of Arc is however strictly timed, abruptly ending within 10 min of the activating input *in vivo*. The resulting bursts of Arc gene transcription are so defined that their detection is widely utilized to map and infer the activity of neuronal ensembles *in vivo* (Guzowski et al., 1999).

Within 15–20 min from the onset of transcription, Arc mRNA is exported to the cytoplasm where it undergoes translation. However, a pool of Arc mRNAs is assembled in neuronal granules and translocates to activated dendrites, a process mediated by a DTE in its ORF and two DTEs in its 3′ UTR (Steward et al., 1998; Kobayashi et al., 2005; Gao et al., 2008; Dynes and Steward, 2012; Farris et al., 2014). Several lines of evidence suggest that, as for many other localized mRNAs, Arc dendritic transport occurs in a translationally silenced state. Known repressors of Arc translation include the FMRP and several miRNAs (Zalfa et al., 2003; Park et al., 2008; Wibrand et al., 2012; Panja et al., 2014). Translational de-repression of Arc in L-LTP has been proposed to act at the initiation step, and is dependent on PKA, ERK, and MNK1 activity (Bloomer et al., 2008; Panja et al., 2009, 2014; Soule et al., 2012). However, in mGluR-dependent LTD, Arc translational activation occurs primarily at the elongation step, modulated by FMRP and by phosphorylation of the elongation factor 2 (eEF2) (Park et al., 2008; Niere et al., 2012). Further, a recent quantitative analysis of an Arc-Gaussia luciferase reporter shows that mGluR-dependent translation of Arc occurs in discrete quantal bursts, induced by the release of stalled polyribosomes on Arc’s coding region (Na et al., 2016). Arc translation is quickly followed by proteasome-mediated decay of the protein, which further limits the time window of Arc protein surge to short bursts of activity-dependent expression (Soule et al., 2012; Pastuzyn and Shepherd, 2017).

Finally, spatial and temporal restriction of Arc expression is also achieved by rapid decay of Arc mRNA upon its translational activation (Giorgi et al., 2007; Soule et al., 2012; Farris et al., 2014; Ninomiya et al., 2016). This ensures that activity-dependent expression of the mRNA decays after 1–2 h from the initial stimulus (Bateup et al., 2013).

One unique feature of Arc mRNA, is the presence of two conserved introns in its 3′ UTR which contribute to its translation-dependent decay by targeting it for destruction via the NMD pathway (Giorgi et al., 2007). Introns are usually positioned within the 5′ UTR or coding regions of eukaryotic genes. Concomitantly to their removal, EJCs are deposited by the spliceosome and facilitate mRNA export and translation (Nott et al., 2004; Gromadzka et al., 2016; Le Hir et al., 2016). Because of the unique positioning of Arc 3′ UTR introns, EJCs are constitutively deposited downstream of Arc mRNA natural stop codon. Upon translational activation, interaction between the Arc 3′ UTR-bound EJCs and the ribosome stalled at the stop codon triggers NMD and degradation of the mRNA (Giorgi et al., 2007).

Confirmation that Arc 3′ UTR splicing downregulates Arc mRNA abundance upon neuronal activation was also recently provided *in vivo* (Steward et al., 2018), adopting EGFP-Arc transgenic mice with an intron-less 3′ UTR region. Additionally, Steward and colleagues find that 3′ UTR splicing is required for efficient dendritic delivery of Arc mRNA.

Post-transcriptional regulation of Arc is thus key in controlling the spatial and temporal confinement of its activity-dependent expression, however the underlying mechanism remains poorly characterized.

In this study, we further investigate how splicing of Arc mRNA 3′ UTR region contributes to modulate Arc expression in neurons. Luciferase reporter constructs harboring Arc 3′ UTR, with or without introns, were transfected in cultured cortical neurons. Remarkably, we find that stimulating transfected cultures with BDNF induces a significant translational upregulation of the reporter, which is dependent on splicing of Arc 3′ UTR introns. Dephosphorylation of eIF2α, mTOR activation, ERK, PKC, and PKA activity participate in the signaling process linking BDNF to splicing-dependent translational upregulation of the Arc 3′ UTR reporter. Further, CREB-dependent transcription is also a prerequisite in this process, suggesting that the molecular cascade involves *de novo* transcription of one or more *trans-*acting factors. Finally, human Arc 3′ UTR confers an even higher translational responsiveness to BDNF, suggesting that splicing-dependent translational upregulation mediated by Arc unique 3′ UTR is conserved and possibly more pronounced in humans. Overall, we find that Arc 3′ UTR splicing induces concurrent mRNA decay and translational upregulation of the mRNA, likely contributing to limit Arc expression to short efficient bursts of expression. This novel regulatory mechanism is relevant to our understanding of EJC function and adds another level of complexity to the expression of this unique effector of synaptic plasticity.

## Results

### Arc 3′ UTR Downregulates Reporter Expression and Recapitulates 3′ UTR Splicing-Dependent NMD

**FIGURE 1:**
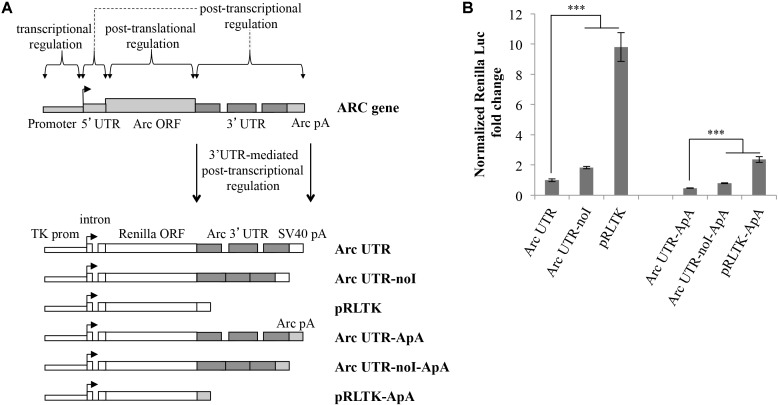
Luciferase constructs harboring Arc 3′ UTR are downregulated and subjected to NMD. **(A)** Schematic representation of the luciferase constructs utilized in this study. Upper panel: schematic Arc gene architecture is depicted, including the general contribution of each region to Arc gene expression. Lower panel: schematic representation of the six Renilla luciferase constructs generated. All constructs are under the control of a Thymidine kinase promoter and include a constitutive intron in the their 5′ UTR. Arc mRNA 3′ UTR was inserted within the pRLTK plasmid (pRLTK) downstream of the Renilla luciferase coding sequence and upstream of SV40 poly(A) sequence (SV40 pA), generating ArcUTR construct. The ArcUTR-noI construct was obtained omitting Arc 3′ UTR introns. SV40 pA was substituted with Arc polyadenylation sequences (Arc pA) to generate Arc UTR-ApA, Arc UTR-noI-ApA, and pRLTK-ApA. **(B)** Luciferase activity of the indicated constructs tested 24 h post-transfection of cortical neurons (8–9 d.i.v.). The histogram reports Renilla luciferase activity normalized to Firefly activity, expressed as fold change compared to Arc UTR. The results are expressed as the mean ± standard error (SEM) from three independent experiments with four biological replicates each (*n* = 12). Statistical significance is calculated relative to Arc UTR or Arc UTR-ApA for each set. Student’s *t*-test: ^∗∗^*P* < 0.01, ^∗∗∗^*P* < 0.001. Multiple comparisons were performed using *t*-tests with Bonferroni correction based on the number of comparisons.

Splicing of Arc 3′ UTR introns participates in modulating stability of this mRNA (Giorgi et al., 2007). To investigate further how this unique 3′ UTR contributes to regulate Arc expression in neurons, we cloned it downstream of the Renilla luciferase ORF and tested reporter expression upon transient transfection in cultured rat cortical neurons. As indicated in **Figure 1A**, the luciferase reporter plasmids we generated harbor different combinations of Arc 3′ UTR elements. Plasmid pRLTK, the parental luciferase expression vector, provides constitutive expression of the Renilla luciferase protein under the TK promoter and SV40 PAS. In plasmid Arc UTR, the genomic rat Arc 3′ UTR sequence was inserted downstream of the Renilla luciferase ORF and upstream of the SV40 PAS. To test specifically how splicing of Arc 3′ UTR affects expression of the reporter, we generated a luciferase plasmid in which both introns were omitted (**Figure 1A**; Arc UTR-noI plasmid) by inserting a cDNA-derived Arc 3′ UTR.

In a parallel set of constructs, SV40 PAS sequences were substituted with Arc PAS (ApA), generating pRLTK-ApA, Arc UTR-ApA, Arc UTR-noI-ApA.

Splicing promotes efficient nucleo-cytoplasmic export and translation of mRNAs (Nott et al., 2004; Le Hir et al., 2016). Hence, all the luciferase constructs described above share a 5′ UTR-encoded chimeric intron, ensuring uniform mRNA cytoplasmic export rates and basal translational efficiency.

The Renilla luciferase constructs described above were utilized to transfect cultured rat cortical neurons (7–9 d.i.v.), in combination with a Firefly luciferase internal control plasmid (pGL3) to normalize for differences in transfection efficiency and culture conditions. 24 h post-transfection, cell lysates were subjected to a dual-luciferase reporter assay, normalizing the Renilla luciferase activity against the Firefly luciferase signal. As shown in **Figure 1B**, insertion of Arc 3′ UTR leads to a sixfold downregulation of the parental construct expression levels (**Figure 1B**; lane Arc UTR versus pRLTK) indicating a general inhibitory activity of this UTR on gene expression. The twofold upregulation observed upon omission of Arc 3′ UTR introns (**Figure 1B**; compare Arc UTR-noI to Arc UTR) confirms that Arc 3′ UTR splicing induces NMD of the Arc UTR reporter, and matches with the twofold increase previously observed on endogenous Arc mRNA upon NMD inhibition (Giorgi et al., 2007). We conclude that our 3′ UTR constructs reproduce the NMD effect observed on endogenous Arc and that other elements in Arc 3′ UTR, in addition to splicing of its introns, contribute to repress expression of the luciferase reporter.

As expected, substituting SV40 strong PAS with those encoded in the rat ARC gene leads to a further downregulation of the reporter expression levels. However, the relative expression levels of the three constructs and the NMD efficiency observed are substantially the same as observed with SV40-driven 3′ end processing. To allow for stronger and more reproducible luciferase signals, all the experiments presented hereafter were performed with the set of constructs encompassing SV40 PAS.

### BDNF Signaling Increases Arc UTR Reporter Expression in a Splicing-Dependent Manner

In the absence of inducing stimuli Arc mRNA and protein are almost undetectable *in vivo* and *in vitro* (Giorgi et al., 2007; Steward et al., 2014, 2018). However, its transcription and translation are strongly enhanced by several plasticity inducing stimuli including exogenous addition of BDNF to *in vitro* neuronal cultures (Bramham et al., 2010; El-Sayed et al., 2011; Leal et al., 2014).

**FIGURE 2:**
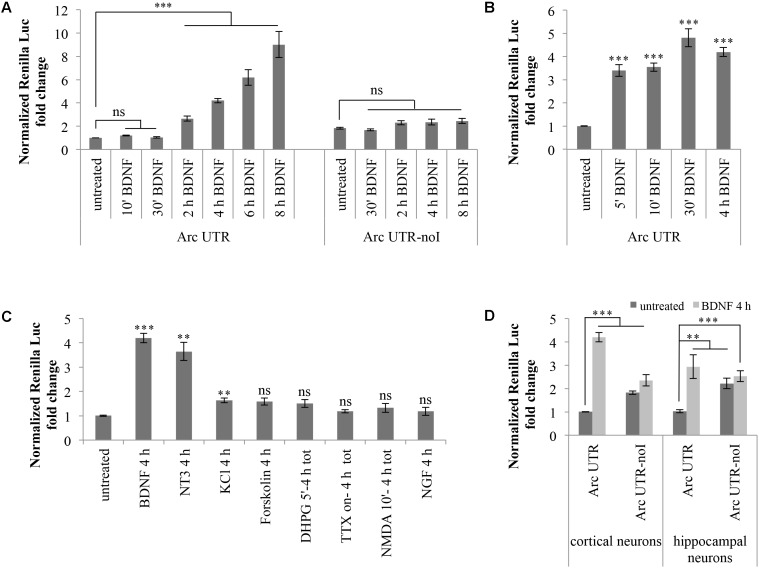
Brain-derived neurotrophic factor induces a significant upregulation of Arc UTR construct, dependent on splicing of its 3′ UTR introns. **(A)** Luciferase assay of cortical neurons transfected with Arc UTR or Arc UTR-noI constructs and subjected to a time course chronic treatment with BDNF (100 ng/ml). **(B)** Luciferase assay of cortical neurons transfected with the Arc UTR construct and incubated with BDNF (100 ng/ml) for 5, 10, or 30 min, followed by thorough washes and incubation in media for a total of 4 h. Luciferase activity obtained upon 4 h chronic BDNF treatment is shown for comparison. **(C)** Luciferase assay of cortical neurons transfected with the Arc UTR construct and treated with the indicated compounds (treatment details, see section “Materials and Methods”). **(D)** Luciferase assay of cortical and hippocampal neurons transfected with the Arc UTR construct and incubated with BDNF (100 ng/ml) for 4 h. **(A–D)** Luciferase assays reporting Renilla luciferase activity normalized to Firefly activity, expressed as fold change compared to the “untreated” sample. All the results are expressed as the mean ± standard error (SEM) from at least three independent experiments with two/three biological replicates each (n between 6 and 43). Student’s *t*-test: ^∗^*P* < 0.05, ^∗∗^*P* < 0.01, ^∗∗∗^*P* < 0.001, ns, non-significant. Multiple comparisons were performed using *t*-tests with Bonferroni correction based on the number of comparisons.

Hence, we investigated whether basal expression of our Arc UTR reporter is affected by BDNF treatement. Cortical neurons (8–10 d.i.v.) transfected with Arc UTR luciferase constructs were chronically treated with 100 ng/ml BDNF for several time points. As shown in **Figure 2A**, this treatment leads to a pronounced upregulation of the Arc UTR Renilla reporter, visible at 2 h of BDNF treatement and persistently increasing up to 10-fold after 8 h of treatment (**Figure 2A**). Interestingly, this positive responsiveness to BDNF was observed only upon transfection of the intron-containing Arc UTR reporter, while the intron-less Arc UTR-noI construct fails to be significantly upregulated even at late time points.

Several forms of LTP induction at glutamatergic synapses require rapid BDNF secretion to elicit both early and late phases in BDNF-dependent LTP (Kovalchuk et al., 2002; Aicardi et al., 2004; Blum and Konnerth, 2005; Edelmann et al., 2014; Panja and Bramham, 2014; Panja et al., 2014; Sasi et al., 2017). We thus tested whether a short and more physiological pulse of exogenous BDNF was able to induce upregulation of our Arc UTR reporter construct. We compared the effects of a 4 h BDNF chronic treatment of Arc UTR-transfected neurons with those obtained by a short induction with BDNF for 5, 10, or 30 min followed by incubation in neuronal media for a total of 4 h. As shown in **Figure 2B**, even a short (5 min) initial pulse with BDNF is sufficient to trigger a significant upregulation of Arc UTR constructs after 4 h of incubation in media, quantitatively comparable to that observed upon 4 h of chronic BDNF treatment.

These results indicate that the 3′ UTR region of Arc mRNA is alone able to confer responsiveness to BDNF, even to a short pulse of the neurotrophin, and that the observed upregulation is dependent on splicing of Arc 3′ UTR introns.

Similar results were obtained transfecting more mature cortical neurons in culture (Supplementary Figure S1A). In particular, 13 d.i.v. cultured neurons were transfected with the Arc UTR and Arc UTR-noI constructs and luciferase signals assayed at 16 d.i.v, prior and upon a 4 h BDNF induction. Luciferase signals were lower, likely due to diminished transfection efficiency of neurons cultured for more than 10 d.i.v. Nontheless, the data shown in Supplementary Figure S1A indicate that our Arc UTR reporter is subjected to NMD and to a splicing-dependent upregulation upon BDNF signaling also in these mature neuronal cultures.

### Other Stimuli, Known to Induce Arc Endogenously, Fail to Reproduce a Significant Upregulation of the Arc UTR Construct Observed With BDNF

We next asked whether the Arc 3′ UTR splicing-dependent upregulation observed upon stimulation with BDNF could be replicated with other protocols known to induce Arc expression in cultured neurons. In particular, we tested whether our Arc UTR reporter levels were affected by: (i) KCl depolarization (Waltereit et al., 2001); (ii) Forskolin application (Bloomer et al., 2008); (iii) NMDARs activation by brief (5 min) bath incubation with NMDA and glycine (Bloomer et al., 2008); (iv) mGluR LTD induction with brief (5 min) DHPG application (Park et al., 2008; Niere et al., 2012); (v) Tetrodotoxin (TTX) withdrawal followed by 4 h washout (Steward et al., 1998; Rao et al., 2006).

Cortical neurons (8–10 d.i.v.) transfected with the Arc UTR luciferase construct were treated with BDNF or with the mentioned compounds for the indicated times (**Figure 2C**). Luciferase levels were then analyzed at 4 h from the beginning of the treatment to gain detectable changes in reporter protein accumulation. As shown in **Figure 2C**, of the induction protocols tested, only KCl depolarization, PKA activation by forskolin and DHPG induction of LTD are able to elicit a very small yet detectable (around 1.5-fold) increase in Arc UTR reporter levels. However, only the increase observed with KCl depolarization is statistically significant and none of these treatments induces an upregulation of the Arc UTR reporter comparable to the one observed with BDNF.

Next, we tested the ability of other neurotrophins, particularly NGF or neurotrophin 3 (NT-3), to replicate the effects observed upon BDNF application. NGF binds to the TrkA receptor and was previously shown to induce Arc expression in PC12 cells while having very limited effects on protein synthesis in neurons (Lyford et al., 1995). NT3, instead, favors neuronal protein translation and binds preferentially to TrkC receptors. However, NT3 can also bind NGF and BDNF cognate receptors TrkA and TrkB, albeit with lower affinity (Ryden and Ibanez, 1996; Huang and Reichardt, 2001; Takei et al., 2001). The effects of these NTs in our assay are reported in **Figure 2C**: while NGF does not affect the luciferase emission of our Arc UTR reporter, incubation with NT3 leads to an increase of its expression comparable to that obtained with BDNF. Overall, of the induction treatments tested, only BDNF and NT3 distinctively induce a strong upregulation of our Arc UTR reporter. These results suggests that Arc 3′ UTR splicing participates in increasing Arc expression levels only upon TrkB, and possibly TrkC, receptor activation.

Finally, we tested whether the splicing-dependent upregulation of our Arc UTR reporter was specific to cortical neurons or could be replicated in hippocampal cultures as well. As shown in **Figure 2D**, expression of the Arc UTR reporter is induced by BDNF in a splicing-dependent manner also in hippocampal neurons. However, the threefold increase obtained at 4 h of chronic BDNF treatment appears less pronounced than the fourfold effect observed in cortical neurons.

### 3′ UTR Splicing Is Necessary but Not Sufficient to Confer Responsiveness to BDNF

**FIGURE 3:**
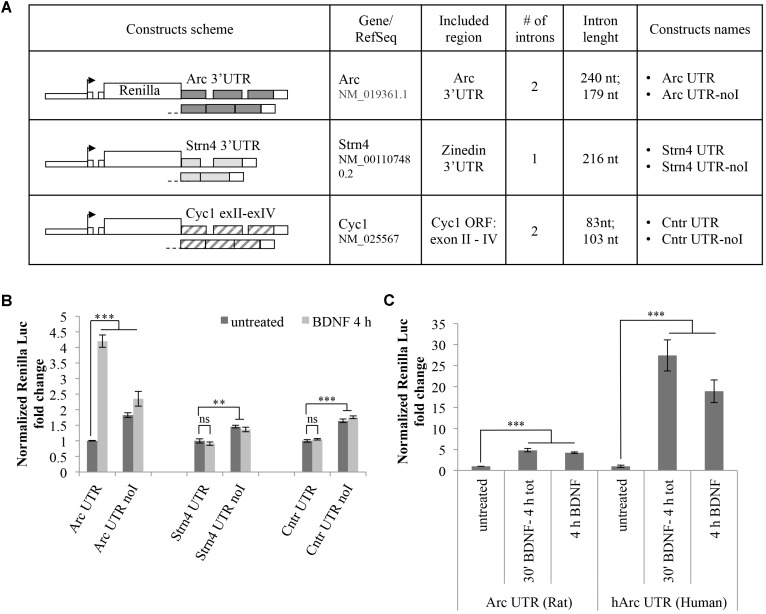
3′ UTR splicing is necessary but not sufficient to confer responsiveness to BDNF. **(A)** Schematic representation of the Renilla luciferase constructs harboring control 3′ UTR regions. The genomic or the cDNA-derived 3′ UTR region of rat STRN4 gene was cloned downstream of the Renilla luciferase coding region generating respectively an intron-containing (Strn4 UTR) and an intron-less control construct (Strn4 UTR noI). Similarly, construct Cntr UTR and Cntr UTR-noI harbor three exons and two small introns of the Cyc1 gene coding region or the corresponding intron-less cDNA fragment downstream of the Renilla ORF. **(B)** Luciferase assay of cortical neurons transfected with the indicated constructs and treated for 4 h with BDNF (100 ng/ml) or left untreated. For each set of plasmids, asterisks denote statistical significance compared to the vehicle-treated intron-containing construct. Renilla luciferase values for the Strn4 and Cntr constructs were normalized to Firefly and expressed as fold change compared to Arc UTR “untreated” sample. Values are the mean ± standard error (SEM) from at least two independent experiments with three replicates (*n* = 6). **(C)** Luciferase signals from rat cortical neurons transfected with the Arc UTR construct or a plasmid harboring the human Arc UTR sequence downstream of the Renilla luciferase gene (hArc UTR). Neurons were either left untreated or incubated with BDNF (100 ng/ml) for 4 h or for 30 min followed by thorough washes and incubation in media for a total of 4 h. Values obtained with Arc UTR construct are reported for comparison and were obtained as indicated in **Figure 2**. Histograms represent Renilla luciferase activity normalized to Firefly activity, expressed as fold change compared to the corresponding “untreated” sample. **(B,C)** Student’s *t*-test: ^∗^*P* < 0.05, ^∗∗^*P* < 0.01, ^∗∗∗^*P* < 0.001, ns, non-significant. Multiple comparisons were performed using *t*-tests with Bonferroni correction based on the number of comparisons.

The observed BDNF-induced upregulation of the Arc UTR reporter is dependent on splicing but it may also require other *cis-*acting elements present in Arc 3′ UTR. To test whether 3′ UTR splicing is sufficient to drive upregulation of the reporter, we modified our luciferase constructs swapping Arc 3′ UTR region with two different intron-containing 3′ UTR sequences and assayed their responsiveness to BDNF (**Figure 3**). In a first set of constructs, we tested the 3′ UTR region of the STRN4 mRNA which, similarly to Arc, is dendritically localized, translated upon NMDARs activation (Lin et al., 2017) and is one of 149 natural substrates for NMD harboring a small conserved intron in its 3′ UTR (Giorgi et al., 2007). Hence, we cloned the genomic or the cDNA-derived 3′ UTR region of rat STRN4 gene downstream of the Renilla luciferase coding region generating respectively an intron-containing (Strn4 UTR) and an intron-less construct (Strn4 UTR noI) (**Figure 3A**).

As an additional set of control plasmids, we substituted the Arc 3′ UTR fragment of our reporter with a portion of the mitochondrial Cytochrome C1 (CYC1) gene containing three exons and two small introns of the coding region (construct Cntr UTR) or the corresponding intron-less cDNA fragment (construct Cntr UTR-noI) (**Figure 3A**). The rationale behind the choice of this control 3′ UTR region is that it is similar in size and number of introns to Arc 3′ UTR and it is constitutively spliced. Additionally, originating from a coding region of an unrelated enzyme it is unlikely to harbor *cis*-acting regulatory elements usually present in the UTR regions of dendritically localized mRNAs. Luciferase expression levels of these constructs were assayed with or without BDNF addition and compared to those obtained with Arc UTR reporters (**Figure 3B**). As expected, for both sets of constructs, the presence of introns in the 3′ UTR decreases the expression levels of the reporter compared to their intron-less counterpart, likely indicative of a NMD effect (**Figure 3B**, compare -UTR vs. -UTR-noI constructs in untreated samples). However, contrary to what observed with Arc UTR constructs, BDNF treatment fails to upregulate the intron-containing STRN4 UTR or the Cntr UTR reporter (**Figure 3B**, compare -UTR untreated vs. -UTR BDNF-treated samples). This suggests that splicing of Arc 3′ UTR *per se* is necessary but not sufficient to elicit expression of the Renilla reporter upon BDNF treatment. Thus, along with splicing of 3′ UTR introns, other elements present in Arc 3′ UTR region are required for the observed upregulation induced by BNDF. This prompted us to test the conservation of this process, swapping the rat Arc UTR region with that of human origin. Interestingly, the human Arc UTR construct is even more efficient in driving upregulation of the reporter upon BDNF addition, bringing Renilla levels up to a 30-fold increase (**Figure 3C**). This entails that human Arc 3′ UTR contains *cis*-acting elements able to amplify this upregulation, and that this process is conserved and potentially more relevant in the human brain than in the rat brain.

### 3′ UTR Splicing-Dependent Upregulation Induced by BDNF Does Not Require Arc 3′ UTR Primary DTE While It Is Affected by Deletion of the Helav/miR-19 Binding Site

Next, we investigated known *cis*-acting elements within Arc 3′ UTR that may contribute to the observed splicing-dependent upregulation. One candidate element we analyzed is the primary 3′ UTR-encoded DTE region which was recently suggested to participate in human Arc mRNA translation-dependent decay in SH-SY5Y cells (Ninomiya et al., 2016).

**FIGURE 4:**
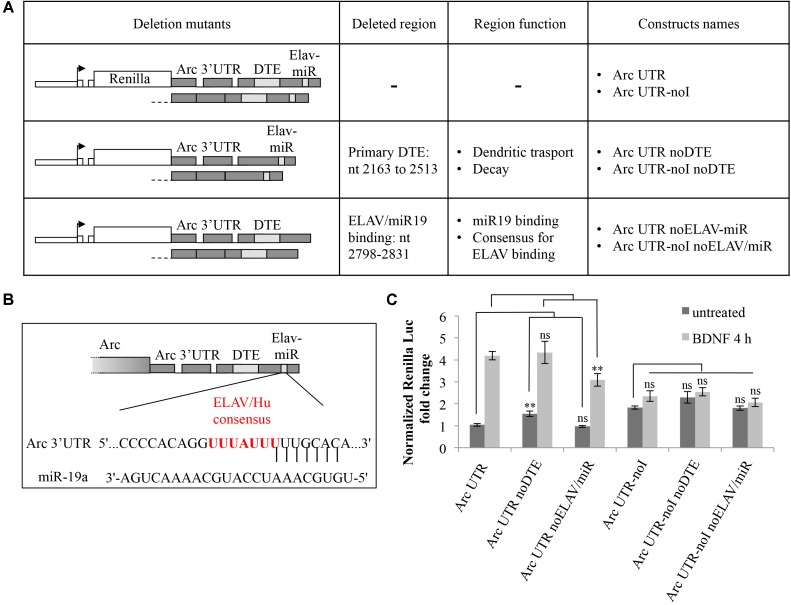
Splicing-dependent upregulation of Arc UTR is not affected by deletion of the primary DTE but is partially inhibited by deletion of a ELAV/miR-19 binding site. **(A)** Schematic representation of the intron-containing and intron-less reporter deletion mutants, in which either the primary DTE or the putative ELAV/miR-19 binding site were deleted. **(B)** Schematic representation of the rat Arc 3′ UTR region targeted by rno-miR-19a (nt 2809–2831 of NM_019361.1), as shown on TargetScan, release 7.1. Vertical lines indicate the seed pairing within the 8mer (Lewis et al., 2005). The overlapping consensus sequence for the ELAV/Hu family of proteins is shown in red. **(C)** Luciferase assay of neurons transfected with the indicated constructs treated for 4 h with BDNF (100 ng/ml) or left untreated. Renilla luciferase values were normalized to Firefly and expressed as fold change compared to Arc UTR “untreated” sample. Bars represent the mean ± standard error (SEM) from at least four independent experiments (n between 9 and 15). Student’s *t*-test: ^∗∗^*P* < 0.01, ^∗∗∗^*P* < 0.001, ns, non-significant. Multiple comparisons were performed using *t*-tests with Bonferroni correction based on the number of comparisons.

Another element in Arc 3′ UTR we sought to investigate is the miR-19a binding site (Wibrand et al., 2012). We noticed (**Figure 4B**) that this highly conserved region encompasses an AU-rich sequence (UUUAUUU at nt 2818–2824), a consensus binding site for the ELAV/Hu family of RNA binding proteins (Kobayashi et al., 2005). ELAV/Hu proteins influence mRNA splicing, stability and translation, colocalize with neuronal dendritic granules, are expressed in an activity dependent manner and can relieve mRNAs from miR-dependent translational repression (Bolognani et al., 2004; Darnell, 2013). Thus, we hypothesized that ELAV/Hu binding to this region could compete with miR-19a and enhance translation of Arc upon BDNF induction. We mutagenized our constructs to specifically delete either the primary DTE or the ELAV/miR-19a binding site in both our intron-containing and intron-less reporters. In **Figure 4A**, the resulting constructs are schematically represented; (i) Arc UTR noDTE and Arc UTR noDTE-noI were deleted of the primary DTE element; (ii) Arc UTR noELAV/miR and Arc UTR-noI noELAV/miR constructs where deleted of a region encompassing the overlapping miR-19 and putative ELAV binding sites. Luciferase assay of neurons transfected with these constructs revealed that deletion of the primary DTE results in a small increase of reporter levels in the uninduced condition, in agreement with data published by Ninomiya et al. (2016). However, removal of the DTE does not affect the response to BDNF induction in a significant way (**Figure 4C**). Further, deletion of the miR/ELAV binding region did not increase expression of the Arc UTR mutant reporter in the uninduced condition, suggesting that Arc 3′ UTR is not a target of miR-19a in these conditions. On the other hand, the miR/ELAV deletion mutant shows a slightly impaired response to BDNF, indicating that this element contributes, albeit marginally, to the splicing-dependent upregulation of Arc UTR reporter induced by BDNF.

### BDNF Modulates Translation, Not Stabilization or Alternative Splicing, of the ARC UTR Reporter

**FIGURE 5:**
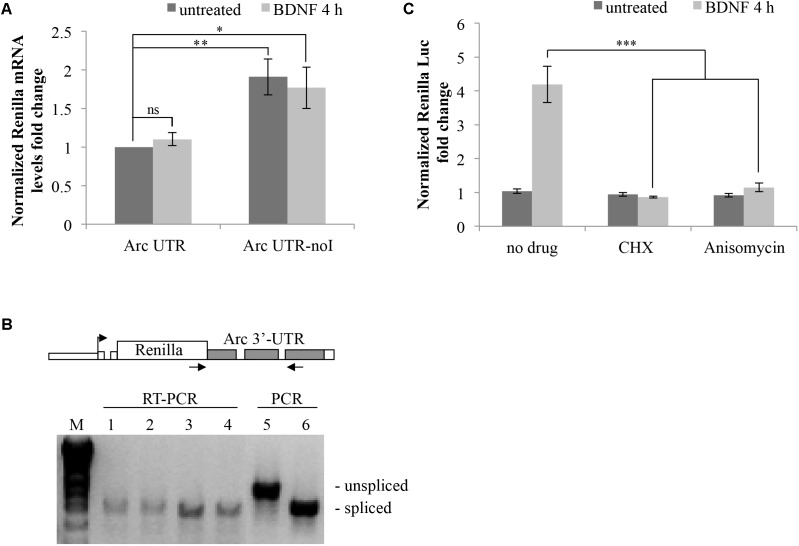
Brain-derived neurotrophic factor promotes translation of Arc UTR reporter while not affecting mRNA stability, splicing pattern or NMD efficiency. **(A)** qRT-PCR analysis of RNAs extracted from neurons transfected with the indicated constructs and treated with BDNF for 4 h or left untreated. Renilla reporter mRNA levels were normalized to Firefly mRNA levels and are expressed as fold change compared to the untreated ARC UTR sample. Bars represent the mean ± standard error (SEM) from 10 biological replicates and two technical replicates each (*n* = 20). Asterisks denote statistical significance compared to the untreated intron-containing construct. **(B)** RT-PCR assay to analyze the splicing pattern of the Arc UTR and Arc UTR-noI reporter mRNAs prior and upon BDNF treatment. (Upper) Schematic representation of the oligonucleotides utilized in the RT-PCR and in the PCR reactions, spanning the Arc UTR intronic region and specific to the Renilla reporter. (Lower) RT-PCR amplification of RNAs extracted from neurons transfected with the Arc UTR (1,2) or the Arc UTR-noI reporter (3,4) and treated with BDNF for 4 h (2,4) or left untreated (1,3). As a size reference of spliced versus unspliced mRNA, RT-PCR products were resolved next to PCR amplifications obtained using Arc UTR (5) or Arc UTR-noI (6) plasmid DNA as template. **(C)** Luciferase assay of neurons transfected with the Arc UTR construct and treated for 4 h with BDNF (100 ng/ml) or left untreated. The indicated drugs (CHX and Anisomycin) were added to the cells 30 min prior to the treatment. Renilla luciferase values were normalized to Firefly and are expressed as fold change compared to Arc UTR “untreated” sample. Error bars represent the standard error (SEM) from at least four independent experiments (n between 8 and 12). Asterisks denote statistical significance compared to the “no drug” sample treated with BDNF for 4 h. Student’s *t*-test: ^∗^*P* < 0.05, ^∗∗^*P* < 0.01, ^∗∗∗^*P* < 0.001, ns, non-significant. Multiple comparisons were performed using *t*-tests with Bonferroni correction based on the number of comparisons.

We then investigated the molecular mechanism underling the observed splicing-dependent upregulation of Arc-UTR constructs upon BDNF treatment. Arc UTR-dependent upregulation of the reporter upon BNDF signaling could be due to stabilization, alternative splicing or enhanced translational efficiency of the intron containing mRNA. To address this, we examined the mRNA levels of the transfected reporters prior or after 4 h of BDNF treatment. Quantitative real-time PCR (qRT-PCR) of the extracted RNAs was performed using a primer set specific for the Renilla coding region, and normalized to Firefly RNA levels. The results shown in **Figure 5A** reveal that, upon BDNF treatment, the overall levels of the Renilla reporter mRNA harboring Arc 3′ UTR are unchanged. Further they confirm that the intron-containing construct undergoes NMD, as its levels are twofold less than the intron-less reporter mRNA. We conclude that the splicing-dependent upregulation we have observed upon BDNF treatment is not due to an increase in transcription rates or in stability of the intron-containing reporter mRNA.

Another possible scenario is that BDNF could trigger alternative splicing or intron inclusion of Arc 3′ UTR region, resulting in the insertion of additional *cis-*elements (e.g., protein-binding sequences) responsible for the observed upregulation. To test this possibility, we examined the splicing pattern of our reporters, subjecting the RNAs extracted from these same cells to RT-PCR with primer pairs spanning the intron containing 3′ UTR region of exogenous constructs (**Figure 5B**). RT-PCR products were resolved next to PCR products, amplified with the same primers but using the reporter plasmid as a template, to compare the size of spliced versus intron-containing PCR products. The 3′ UTR RT-PCR fragments obtained from cells transfected with the intron-containing Arc UTR or with the intron-less Arc UTR-noI constructs co-migrate independently of BDNF addition. This indicates that the Arc UTR region in our reporter is fully spliced and does not undergo alternative splicing or intron inclusion upon BDNF induction. The same result was obtained analyzing the splicing pattern of endogenous Arc mRNA in untransfected neurons that were induced with BDNF or left untreated (Supplementary Figure S1B).

We conclude that BDNF affects translation, not transcription, stability, or alternative splicing of our Arc UTR construct. In support of this scenario, blocking translation by pretreating Arc UTR transfected neurons with low levels of anysomycin or cycloheximide prior to BDNF induction completely abolishes the observed upregulation of the intron-containing reporter (**Figure 5C**). Notably, this treatment only slightly affects overall reporter protein levels (data not shown) and normalization to Firefly protein synthesis controls for this small general decrease. Thus, BDNF leads to a translational upregulation of the fully spliced Arc UTR reporter.

### BDNF Increases Translational Efficiency of ARC UTR Reporter in a Splicing-Dependent Manner

To further corroborate this evidence, we monitored the translational activity of our reporters in a polysome profile assay.

**FIGURE 6:**
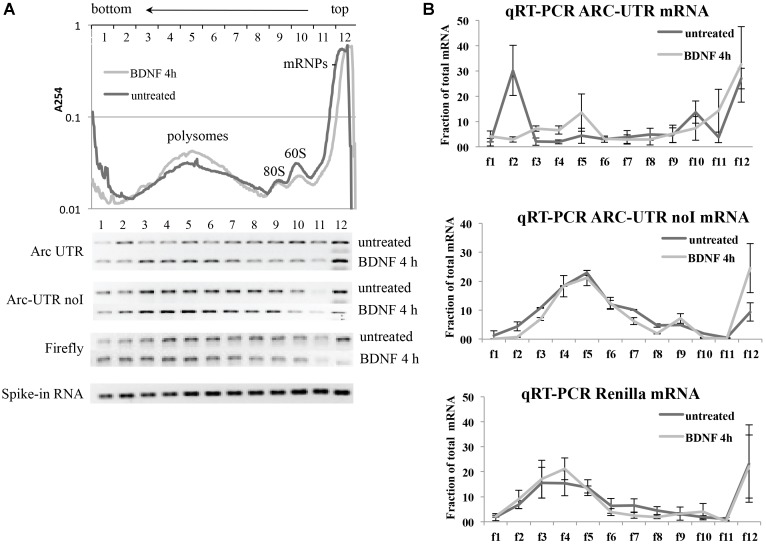
Brain-derived neurotrophic factor treatment increases the association of ARC UTR reporter with translating polyribosomes in a splicing-dependent manner. (**A**; upper panel) Representative polysome profiles of rat cortical neurons co-transfected with the Firefly Luciferase construct and with Arc UTR or Arc UTR-noI Renilla constructs and incubated with BDNF for 4 h or left untreated. Cytosolic lysates were separated on a 15–50% sucrose gradient to separate mRNPs, monosome and polysome fractions. Twelve fractions were collected with UV monitoring of RNA levels at A254. Fraction numbers are indicated at the top of the panel. RNAs from each fraction were extracted together with a spike-in RNA transcript to control and normalize for recovery efficiency. RNAs were then subjected to real time RT-PCR to detect the relative distribution on the polysome gradient of our Renilla reporter mRNA as a consequence of splicing and upon BDNF treatment. (**A**; lower panel) qRT-PCR of the indicated reporter mRNAs recovered from each fraction was resolved on agarose gel to visualize their distribution along the sucrose gradients before and after BDNF treatment. The bottom lane shows a representative qRT-PCR of the spike-in RNA recovered from each polysomal fraction and utilized to normalize qRT-PCR results. **(B)** Quantitative RT-PCR was used to measure Arc UTR, Arc UTR-noI, and Firefly mRNA levels from each fraction of the polysome gradients, as described in Section “Materials and Methods.” Profiles from neurons treated with BDNF (light gray) or left untreated (dark gray) are superimposed. Data are plotted as a fraction of the total recovered from the gradient, and are normalized for spike-in RNA recovery from each fraction. Error bars represent the standard error (SEM) from two independent polysomes gradients for each transfected reporter and three technical replicates (*n* = 6).

Neurons transfected with either Arc UTR or Arc UTR-noI constructs were left untreated or incubated with BDNF for 4 h. A Firefly construct was also co-transfected to control for overall changes in translational efficiency induced by BDNF. At the end of the treatment, neuronal lysates were separated on a 15–50% sucrose gradient and 12 fractions collected (**Figure 6A**, upper panel). Spike-in RNA was added to each fraction prior to RNA extraction to normalize for differences in RNA recovery. Renilla and Firely mRNA levels in each fraction were then quantified by qRT-PCR of the extracted RNA, normalized against the spike-in RNA recovered in that fraction and plotted as a fraction of the total mRNA (**Figure 6B**). The qRT-PCR reactions were also run on agarose gel to visualize the approximate distribution of the various reporter mRNAs along the gradient (**Figure 6A**, lower panel). The results reveal that neither Firefly nor ARC UTR-noI mRNAs are affected by BDNF in their distribution along the gradient fractions (**Figure 6B**, central and lower panel). On the other hand, we could detect a significant shift in ARC UTR reporter distribution upon BDNF induction (**Figure 6B**, upper panel); in untreated cells, this reporter mRNA co-sediments with the mRNPs (fraction 12), with the 80/60S peaks (fractions 9–10) and with a heavy non-polysomal fraction (fraction 2). Instead, in BDNF treated neurons, ARC UTR reporter co-sediments with the mRNPs, 40S and 60S light fractions (fractions 9–12) and with polysomes (fractions 3–6), similarly to its intron-less counterpart. The peak of Arc UTR reporter from untreated samples detected in the heavy fraction 2 may be indicative of its association with translationally repressed dendritic granules (Shiina et al., 2005). Overall, BDNF treatment appears to affect the translational status of our Arc UTR reporter, suggesting a translational de-repression and enhanced polysome-association that are dependent on splicing.

### The Signaling Cascade Involved in the Splicing-Dependent Translational Upregulation of Arc Upon BDNF Treatment

We next investigated the underlying molecular cascade by inhibiting signaling pathways known to contribute to Arc translational regulation.

Arc translation is tightly controlled by activity through several signaling cascades, depending on the type of inducing stimuli and cellular context (Bramham et al., 2010). In cultured neurons, stimulated NMDARs and GPCRs elicit Arc translation in a manner dependent on PKA (Bloomer et al., 2008). mGluR-LTD depends on translational de-repression of pre-existing Arc mRNA mediated by phosphorylation of the elongation factor eEF2 (Park et al., 2008). In the DG, LTP consolidation depends on Arc translation induced by BDNF signaling to ERK and downstream activation of MNK1, independently of mTOR (Panja et al., 2009, 2014; Panja and Bramham, 2014). However, in cortical synaptoneurosomes BDNF induces translation of dendritic mRNAs (Schratt et al., 2004), including Arc, via activation of the PI3K-mTOR pathway, which favors eIF4F complex formation and S6K1 activation. In this *in vitro* model BDNF translational activation of Arc also requires NMDARs activity (Yin et al., 2002). Further, in cortical synaptoneurosomes the JNK pathway has been shown to regulate mGluR-induced translational de-repression of FMRP targets. Recent data also suggest that Arc translational upregulation may be affected by dephosphorylation of the initiation factor eIF2α (Ma et al., 2013), which is key to protein synthesis-dependent long-term memory formation (Costa-Mattioli et al., 2007; Trinh and Klann, 2013).

Finally, mAChRs activation leads to strong transcriptional (PKC- and ERK-dependent) and translational (ERK-dependent) induction of Arc (Teber et al., 2004; Soule et al., 2012).

In light of these evidences, we tested the involvement of these signaling pathways in coupling BDNF induction to Arc UTR splicing-dependent translational upregulation. Prior to BDNF application, we incubated Arc UTR-transfected neurons with the following inhibitors for the indicated kinases/receptors: U0126 (ERK), cgp57380 (MNK), H89 (PKA), GF109203X (PKC), A484954 (eEF2K), Ku63794 and Rapamycin (mTOR), PF4708671 (S6K1), MK8012 (NMDAR), SP600125 (JNK), Salubrinal (eIF2α dephosphorylation).

**FIGURE 7:**
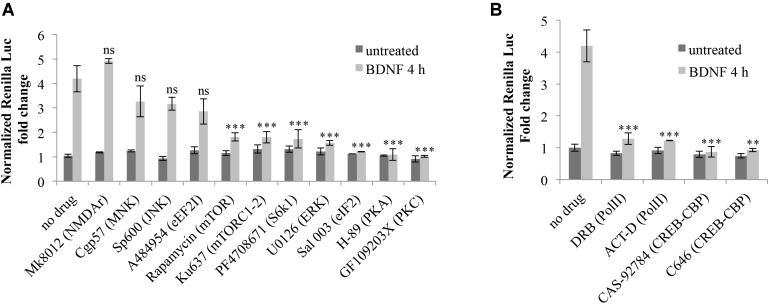
Splicing-dependent translational upregulation of ARC UTR constructs requires the activity of ERK, PKA and PKC kinases, the mTOR/S6K1 pathway, eIF2α dephosphorylation and CREB-dependent transcription. **(A,B)** Luciferase assay of neurons transfected with the Arc UTR construct and treated for 4 h with BDNF (100 ng/ml) or left untreated. Inhibitors for the indicated kinases or receptors or transcription factors were added to the cells 30 min prior to treatment with BDNF or with vehicle. Treatment detail in Section “Materials and Methods.” Renilla luciferase values were normalized to Firefly and are expressed as fold change compared to Arc UTR “no drug – untreated” sample. Error bars represent the standard error (SEM) from at least three independent experiments (n between 6 and 14). Asterisks denote statistical significance compared to the “no drug” sample treated with BDNF for 4 h. Student’s *t*-test: ^∗∗^*P* < 0.01, ^∗∗∗^*P* < 0.001, ns, non-significant. Multiple comparisons were performed using *t*-tests with Bonferroni correction based on the number of comparisons.

The resulting luciferase assays revealed that none of the inhibitors tested significantly alter the steady state expression levels of Arc 3′ UTR construct in the absence of induction (**Figure 7A**). However, upon BDNF addition, Arc UTR splicing-dependent translational upregulation is significantly affected by inhibition of the mTOR/S6K1 pathway, of eIF2α dephosphorylation and of the ERK, PKA, and PKC kinases (**Figure 7A**). This suggests that translation of the Arc UTR reporter may be directly modulated by these kinases and translation factors. Indeed, they all converge on regulating translation initiation. However, another common target of PKA, ERK, PKC and eIF2α, downstream of BDNF, is the activation of CREB (Roberson et al., 1999; Bramham et al., 2010; Trinh and Klann, 2013) a transcription factor critical for synaptic plasticity and long-term memory formation (Alberini and Kandel, 2014).

Hence, it is plausible that the requirement for these kinases may be dependent on their ability to activate CREB-dependent transcription. We thus investigated whether the translational upregulation mediated by Arc 3′ UTR splicing requires CREB activation downstream of BDNF induction. To this end, we utilized actinomycin D or DRB, to inhibit Pol II transcription, and CAS-92784 or C646 to inhibit CREB activity. As shown in **Figure 7B**, inhibition of either Pol II or CREB completely abolishes Arc UTR translational upregulation upon BDNF induction. Hence, CREB-dependent transcription is a necessary step in the signaling cascade liking BDNF to Arc 3′ UTR-dependent translational upregulation.

## Discussion

The 3′ UTR region of messenger RNAs plays a central role in coordinating their post-transcriptional regulation, recruiting RNA binding factors that affect their localization, stability, and translation (Giorgi and Moore, 2007; Gebauer et al., 2012; Matoulkova et al., 2012). Arc mRNA 3′ UTR is particularly interesting as it harbors two conserved introns, which regulate its abundance targeting it for destruction via the NMD (Giorgi et al., 2007; Steward et al., 2018). This feature is extremely unusual among eukaryotic genes and its implications in Arc gene expression have not been fully elucidated. To further investigate how splicing of Arc 3′ UTR contributes to Arc transient expression, we transfected cultured neurons with luciferase reporter constructs harboring Arc 3′ UTR and tested their response under inducing stimuli. This approach allowed us to characterize an additional and unexpected contribution of this unique region to Arc dynamic gene regulation. We find that, upon BDNF induction, splicing of Arc 3′ UTR not only induces mRNA decay via NMD but also elicits translational upregulation of the mRNA. This process is strongly triggered only by BDNF or NT3 signaling and much less, if at all, by other protocols known to induce Arc. We also find that splicing of Arc 3′ UTR is necessary but not sufficient to trigger the translational upregulation, indicating that other elements in the 3′ UTR contribute to this process. Hence, we tested the potential involvement of other elements residing in Arc 3′ UTR. Deletion of a region encompassing the miR-19 binding site, and a coincident putative consensus binding site for the ELAV/Hu proteins, slightly inhibits Arc UTR translational upregulation induced by BDNF while leaving reporter levels unaffected in untreated samples. This suggests that, at least in untreated neurons and in these experimental conditions, Arc 3′ UTR is not targeted by miR-19. However, upon BDNF signaling, this region may recruit miR-19 and/or an ELAV/Hu protein, contributing to facilitate translation of the spliced reporter. Further analyses are needed to test this hypothesis, verifying the binding of a ELAV/Hu protein to Arc 3′ UTR and its interplay with miR-19, with 3′ UTR splicing and with BDNF signaling.

Detection of our Arc UTR reporter mRNA levels by qRT-PCR, and distribution on polysome gradients, revealed that the observed upregulation occurs at the translational level. Upon BDNF treatment, we observe an enhanced association of the spliced Arc UTR reporter mRNA with translating ribosomes while the distribution of the intron-less and control reporter mRNAs along the gradient is unaffected.

**FIGURE 8:**
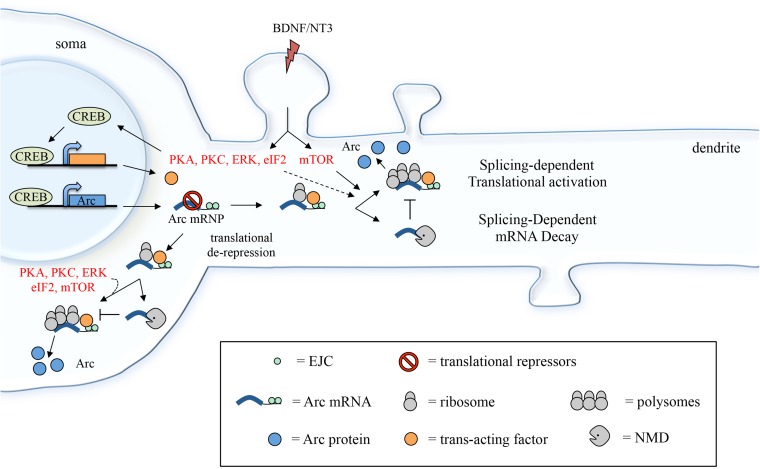
Suggested model depicting how 3′ UTR splicing may participate in fine-tuning Arc activity-dependent expression. Induction with BDNF (or NT3) leads to CREB activation via PKA, PKC and ERK signaling and eIF2α dephosphorylation. CREB, in turn, induces transcription of target genes, including Arc and a putative *trans-*acting factor required to couple Arc 3′ UTR splicing and EJC deposition with translational activation. Following export to the cytoplasm and translational de-repression, Arc mRNA undergoes a first round of translation, eliciting NMD of the mRNA (splicing-dependent mRNA decay). A pool of Arc mRNA “escapes” NMD and the 3′ UTR-bound EJCs mediate translational activation of these transcripts. This process may be directly modulated by mTOR, eIF2α dephosphorylation, PKA, PKC, and ERK and likely requires a CREB-dependent *trans-*acting factor (splicing-dependent translational activation; see section “Discussion”).

This finding was unanticipated as mRNAs that are targeted by NMD undergo mRNA decapping/deadenylation and concurrent translational silencing (via inhibition of the initiation step) (Isken et al., 2008; Schweingruber et al., 2013). It was thus surprising to find that Arc 3′ UTR reporter can be targeted by NMD and nonetheless concomitantly undergo translational activation. This incongruity may be due to the fact that Arc is not a typical NMD substrate: a misprocessed or mutant transcript targeted for destruction due to the erroneous insertion of a premature stop codon. Rather, Arc is one of a few natural substrates of NMD (Giorgi et al., 2007) and may have evolved to partially hijack the NMD pathway as a means to curb its expression while still allowing for a timed (yet efficient) expression under plasticity inducing stimuli. Hence, a possible explanation is that only a subset of Arc mRNAs are targeted by NMD, as indicated by the limited, twofold, increase in mRNA levels observed upon NMD inhibition (Giorgi et al., 2007) or in the absence of splicing. Those Arc mRNA molecules that escape NMD could be exposed to a different type of post-transcriptional regulation in response to BDNF, one in which 3′ UTR splicing enhances, rather than repress, translation (**Figure 8**). It is indeed widely accepted that NMD is not a uniform and constitutive process. To the contrary, its efficiency in eliciting mRNA decay or translational activation varies greatly depending on the cellular context, on the target mRNA sequence and structure and on the composition of the associated EJCs (Gehring et al., 2005; Singh et al., 2008; Muhlemann and Jensen, 2012; Joncourt et al., 2014; Le Hir et al., 2016).

The exon junction complex consists of a core of three proteins, namely eIF4AIII, Y14 and Magoh, which function as an anchor for the binding of EJC accessory factors that mediate its roles in mRNA surveillance, localization, and translation. Accessory EJC factors include, among many others, Upf2, Upf3, and Mln51 and their association to the EJC is dependent on their relative abundance, cellular context and mRNA sequence. Upf2 and Upf3 mediate the EJC’s ability to trigger NMD, via interaction with the stalled ribosome and Upf1. Mln51, on the contrary, is involved in facilitating translational initiation of EJC bound mRNAs by recruiting the initiation factor eIF3b (Chazal et al., 2013). Until recently, Mln51 was considered a ubiquitous core EJC binding factor; however, its low abundance suggests that it may be present only in a subset of EJC complexes (Singh et al., 2015; Mao et al., 2017). Nonetheless, a biochemical characterization of dendritic granules components revealed that Arc mRNA always co-purifies with Mln51-containing granules (Fritzsche et al., 2013) suggesting that this EJC factor is stably associated with Arc mRNA and can facilitate its translational activation.

Overall, a possible scenario is that Arc 3′ UTR splicing leads to deposition of EJC complexes that induce NMD with limited efficiency allowing for a pool of Arc transcripts to escape decay and remain capped and polyadenylated. Upon BDNF signaling, translation of this pool of Arc mRNAs is elicited in a splicing-dependent manner and likely involves EJC-bound Mln51’s ability to activate translation initiation (**Figure 8**). Hence, BDNF may function as a switch to turn on splicing-dependent translation of Arc, either altering EJC composition directly or affecting its ability to activate translation. The molecular mechanism underlying this switch remains to be determined. In this regard, our data shows that, along with splicing, other elements of Arc 3′ UTR are required for the translational activation observed upon BDNF induction. Further, CREB-dependent *de novo* transcription induced by BDNF is also a prerequisite. These results suggest that the molecular switch provided by BDNF involves transcription of a *trans*-acting factor that intervenes in coupling splicing (i.e., the EJC) of Arc with translational activation. Binding of such a *trans-*acting factor (whether a protein or a non-coding RNA) to Arc 3′ UTR would also explain the requirement for other *cis-*elements within this region.

Further analyses are required to elucidate the fine molecular mechanism linking BDNF signaling to the splicing-dependent translational activation of Arc. Particularly the biochemical characterization of Arc 3′ UTR-borne RNA-protein complexes before and after BDNF induction would be instrumental to test the model proposed above and address some of the questions that this study exposes.

The indication that 3′ UTR splicing is not merely a trigger for NMD but can exert a positive effect on gene expression is a novel and unexpected finding, relevant to our understanding of mRNA metabolism and widening our perspective on the role of splicing in defining a gene’s post-transcriptional regulation. It would thus be of interest to understand whether this mechanism is limited to Arc mRNA complex and unique metabolism, or adopted by other mRNA substrates.

The approach utilized in this study was designed to investigate specifically the contribution of Arc unique 3′ UTR region to Arc post-transcriptional regulation under inducing stimuli. Consequently, a limitation of this study is that it does not address how this region affects overall Arc mRNA metabolism and its interplay with other regulatory elements. One such region of interest is the 5′ UTR, which has been reported to contain an IRES element (Pinkstaff et al., 2001) and may likely cooperate with the 3′ UTR-mediated splicing-dependent regulation in fine-tuning Arc translation.

The subcellular localization of this translational modulation is another aspect that necessitates further investigation, particularly in light of recent evidences that link Arc 3′ UTR splicing with its proper dendritic localization (Steward et al., 2018).

An additional question that emerges from this study regards the timing of the observed BDNF-dependent translational up-regulation mediated by Arc 3′ UTR splicing. Arc-UTR luciferase reporter emission increases significantly only after 2 h from BDNF addition, a timing potentially at odds with the limited half-life of endogenous Arc mRNA (45 min). This raises the question of whether endogenous Arc mRNAs, newly-transcribed upon BDNF induction, persist long enough in the cell to be affected by the translational regulation mechanism we described. The 2 h delay we observed could be partially explained by the time required for the Renilla luciferase protein to be translated and folded properly. However, since CREB-dependent expression of a *trans-*acting factor is required for the translational upregulation mediated by Arc 3′ UTR splicing to occur, it is conceivable that this process may affect only the end-tail of the initial burst of Arc expression. On the other hand, once triggered, this process may effectively potentiate translational efficiency of subsequent waves of Arc mRNA, either induced by repetitive stimuli on the same neuron or linked to the biphasic Arc expression observed during memory consolidation *in vivo* (Seibt et al., 2012; Nakayama et al., 2015).

Overall, this study discloses a novel mechanism of post-transcriptional gene regulation in neurons, where Arc 3′ UTR splicing induces concurrent mRNA decay and translational upregulation of the mRNA, contributing to limit Arc expression to short yet efficient bursts of expression. Our findings add to the already multilayered picture of Arc gene-expression, a uniquely complex process aimed at facilitating its role in the activity-dependent modifications of synapses that underlie memory storage.

## Materials and Methods

### Neuronal Cultures

Rat cortical cultures were prepared from embryonic day 17–19 fetal Wistar rats. The brains were removed and cortices were freed of meninges, washed with Earl’s balanced salt solution, and centrifuged for 2 min at 150 × *g*. The tissue was resuspended and incubated for 30 min at 37°C with 0.02% trypsin followed by addition of DNase I (80 μg/mL final) and trypsin inhibitor (0.52 mg/mL). Digested tissues were mechanically dissociated twice and centrifuged at 150 *g* for 10 min. The dissociated cells were plated at a density of 150,000 cells/cm^2^ on wells treated with poly-L-lysine (Sigma-Aldrich) in Neurobasal media (Gibco, cat. no. 21103- 049) supplemented with 2% B27 (Gibco, cat. no. 17504-044), 0.5 mM L-glutamine (Euroclone, cat. no. ECB3000D), and 50 U/mL penicillin/streptomycin solution (Euroclone, cat. no. ECB3001D). Cells were grown at 37°C and 5% CO_2_, changing half medium every 3 days from the seeding to the execution of the experiments. Neurons were transfected between 7 and 9 d.i.v. in all experiments except in one case, when neurons were transfected at 13 d.i.v. and luciferase signals assayed at 16 d.i.v.

All animal procedures were carried out in accordance with the guidelines established by the European Communities Council (Directive 2010/63/EU of 22 September, 2010) and by the Italian Ministry of Health (D.L.116/92) upon approval by the Italian Ministry of Health Ethical Committee “Head Office for Animal Health and Veterinary Drugs.” Approval No.: 527/2017-PR.

### Cloning

Plasmid pRLTK was modified to allow for the insertion of 3′ UTR sequences downstream of the Renilla coding region were a XbaI restriction site resides. A synthetic DNA fragment encompassing XbaI-SalI-XhoI-XbaI sites was inserted within the XbaI-digested pRLTK, generating pRLTK-SX. To generate plasmid Arc UTR, the genomic rat Arc 3′ UTR sequence was PCR amplified from rat genomic DNA with primers rArcU-1 and rArcU-2 and inserted SalI-XhoI in pRLTK-SX upstream of the SV40 polyadenylation element. For plasmid Arc UTR-noI, the intron-less cDNA insert was generated by RT-PCR of total rat RNA, using the same primers and cloning sites as for Arc UTR. In plasmid Strn4 UTR, the genomic rat STRN4 (Zinedin) 3′ UTR sequence was PCR amplified from rat genomic DNA with primers rSTRN4-1 and rSTRN4-3 and inserted SalI-XhoI in pRLTK-SX upstream of the SV40 polyadenylation element. For plasmid Strn4 UTR-noI, the intron-less cDNA insert was generated by one-step RT-PCR of total rat RNA, using the same primers and cloning sites as for Strn4 UTR. To generate plasmid Cntr UTR, a fragment of the genomic mouse CYC1 coding region, spanning exon II-III-IV, was PCR amplified from mouse genomic DNA with primers mCYC1-1 and mCYC1-2 and inserted SalI-XhoI in pRLTK-SX upstream of the SV40 polyadenylation element. In plasmid Cntr UTR-noI, the corresponding intron-less cDNA insert was generated by one step RT-PCR of total mouse RNA, using the same primers and cloning sites as for Cntr UTR.

Plasmid hArc UTR was generated by PCR amplification of human (SH-SY5Y DNA) genomic DNA with primers hArcU-1 and hArcU-2. The purified and XbaI-digested insert was cloned in pRLTK-SX upstream of the SV40 polyadenylation element.

In another set of constructs, the SV40 PAS sequence was substituted with Arc PAS sequence, generating Arc UTR-ApA, Arc UTR-noI-ApA. To this end, a 1128 nt fragment encompassing Arc third exon and PAS elements was amplified from rat genomic DNA using primers 3ExArc-fw and Arc-PAS-rw. The PCR product was then digested KpnI and BamHI and inserted in the corresponding restriction sites of Arc UTR or of Arc UTR-noI constructs, generating Arc UTR-ApA and Arc UTR-noI-ApA respectively. The control pRLTK-ApA plasmid was obtained by PCR amplifying the PAS sequence of Arc 3′ UTR from rat genomic DNA with primers Arc-PAS-fw and Arc-PAS-rw. The 342 nt-long insert was then cloned in the XbaI/BamHI sites of the pRLTK parental vector.

Plasmids Arc UTR and Arc UTR-noI were deleted of the primary DTE element (nt 2163–2513 of NM_019361.1) to generate Arc UTR noDTE and Arc UTR-noI noDTE respectively. Delition was obtained via PCR-based site-directed mutagenesis by overlap-extension (Heckman and Pease, 2007). Briefly, two PCR products (A and B), flanking the deletion, were generated using primers with a terminal overlapping sequence. Primer set A-DTE fw; A-DTE rw and primer set B-DTE fw; B-DTE rw were used to generate product A and B respectively, using both Arc UTR and Arc UTR-noI plasmids as template. The two products were then gel purified, annealed to each other and PCR amplified with the external primers A-DTE fw and B-DTE rw, generating a full-length insert deleted of the targeted region. This insert was then digested SalI and BamHI and reinserted in the corresponding sites of Arc UTR and Arc UTR-noI plasmid.

Deletion of the ELAV/miR-19 binding site (nt 2798–2831 of NM_019361.1) was obtained generating two PCR products flanking this target sequence with an internal overlapping extension that includes a SpeI restriction site. The two PCR products were obtained with primer set A (DMIR19 rw; 3ExArc-fw) and with primer set B (DMIR19 fw; rArcU-2). Arc UTR plasmid was used as template. Product A was digested with KpnI and SpeI, product B was digested with SpeI and XhoI. The purified digested fragments were then ligated together with Arc UTR (or Arc UTR-noI plasmid) digested with KpnI and XhoI, generating Arc UTR noELAV-miR and Arc UTR-noI noELAV/miR. All PCR reactions were performed with High Fidelity Paltinum Taq Polymerase (Invitrogen) and inserts were fully sequenced.

Oligonucleotides used are as follows (restriction sites are highlighted):

rArcU-1 TTTTTTGTCGACACACCCAGTCTGTGGCTTTT;
rArcU-2 TTTTTCTCGAGTGTCTTTGAGGTAAGATGGTGTG;
rArcU-3 TTTTTCTCGAGCACTAGAGCTCCAGCACCAT;
rArcU-4 CCTCTCAGGGTGAGCTGAAG;
rArcU-5 GTCTCCTGGGACTGGACTTG;
rArcU-6 TGCCTTGAAAGTGTCTTGGA;
rSTRN4-1 TTTTTGTCGACTCCCACCTGGCCTCGCC;
rSTRN4-2 TTTTTCTCGAGGAGACAATTTTTTTCACATACAAGTTGGG;
mCYC1-1 TTTTTTGTCGACTCCTTGTCCTCGAAGTCTGG;
mCYC1-2 TTTTTTCTCGAGAGCTCGAACGATGTAGCTGA;
hArcU-1 TTTTTTCTAGAAGGGCATCCCGGAGCC;
hArcU-2 TTTTTTTCTAGACTCTGGGGTAAGGTGCACAG;
3ExArc-fw AAGTCTTTCCGGCCATGTCT;
Arc-PAS-fw TATATATCTAGACTGCCCACACCATCTTACCT;
Arc-PAS-rw GGGCTAGATGAGCCCAGTTC;
A-DTE-fw TTCGTTGAGCGAGTTCTCAAA;
A-DTE-rw CAGGCTGGGCTAGGGCCCAGACTTCTCAGCAGCTTGAGAC;
B-DTE-fw GTCTCAAGCTGCTGAGAAGTCTGGGCCCTAGCCCAGCCTG;
B-DTE-rw GTTATTGTCTCATGAGCGGATACA;
DMIR19-fw AAAAAACTAGTCCATGACCCATACTAATTTGG;
DMIR19-rw AAAAAACTAGTACAGGGTGGGGGATCTGT;
pRLTK-fw GATAACTGGTCCGCAGTGGT;
pRLTK-rw ACCAGATTTGCCTGATTTGC;
Firefly-fw TCAAAGAGGCGAACTGTGTG;
Firefly-rw GGTGTTGGAGCAAGATGGAT;
GFP-fw CACATGAAGCAGCACGACTT;
GFP-rw CTACTTGTACAGCTCGTCCATGC;
pRLTK-ORF TTATTGAATCGGACCCAGGA.

### Transient Transfection and Luciferase Assay

Prior to transfection, pRLTK Renilla luciferase plasmids were carefully quantified with a spectrophotometer and by separation on agarose gel to ensure that equal amounts and same quality of plasmid DNA were transfected. Transfections were performed on 7–9 d.i.v. (or 13 d.i.v.) cultured neurons with Lipofectamine 2000 according to the manufacturer’s instructions. For luciferase assays, neurons were cultured in 48-well-plates and, for each well, the transfection mixture included: 150 ng PRLTK construct, 150 ng pGL3 control plasmid, 200 ng pBlueScript, 0.4 μl Lipofectamine 2000, and 12 μl Opti-MEM medium. Complexes were allowed to form for 20 min at RT. In the meantime, the neuronal medium from each well was removed, stored, and replaced with Neurobasal medium. The transfection mixture was then added to each well and incubated for 4 h at 37°C. Finally, the transfection mixture was removed and the stored neuronal medium was added back to the cultures. After 24 h, transfected neurons were pharmacologically treated (were indicated), lysed and subjected to dual luciferase assay (Promega) according to the manufacturer’s instructions. For RT-PCR and qRT-PCR analyses of reporter mRNA, neurons were cultured in 6-well plates scaling the transfection mixture accordingly.

### Pharmacological Treatments

Treatment of transfected neurons was performed as follows: human recombinant BDNF (100 ng/ml for the indicated times; Società Italiana Chimici); recombinant NT3 and NGF (100 ng/ml for 4 h; generous gifts of Marco Canossa and Francesca Malerba, EBRI); Forskolin (50 μM for 4 h; Sigma Aldrich); KCl (50 mM, 4 h); DHPG (100 μM for 5 min followed by washes and incubation in neuronal media for 4 h; Sigma Aldrich); TTX (2 μM for 24 h followed by washes and incubation in neuronal media for 4 h; Tocris); NMDA (50 μM)/glycine (2 μM) (10 min followed by washes and incubation in neuronal media for 4 h; Sigma Aldrich).

To test the signaling pathways involved, inhibitor compounds were added to the cells 30 min prior to treatment with vehicle (DMSO) or with BDNF. Inhibitors were used as follows: U0126 (ERK inhibitor, 10 μM, Tocris); CGP-57380 (MNK inhibitor, 50 μM, Tocris); H-89 (PKA inhibitor, 10 μM, Sigma Aldrich); GF109203X (PKC inhibitor, 3 μM, Selleckchem), A484954 (eEF2K inhibitor, 10 μM, Calbiochem); Ku63794 (mTORC1 and mTORC2 inhibitor, 5 μM, Selleckchem); Rapamycin (mTOR inhibitor, 2 μM, Tocris); PF4708671 (S6K1 inhibitor, 20 μM, Selleckchem); MK-801 Hydrogen Maleate (NMDAR antagonist, 10 μM, Sigma Aldrich); SP600125 (JNK inhibitor, 1 μM, Tocris), Salubrinal (Sal003, eIF2α dephosphorylation inhibitor, 10 μM, Sigma Aldrich); Anisomycin (protein synthesis inhibitor, 40 μM, Sigma Aldrich); CHX (protein synthesis inhibitor, 10 μg/ml, Sigma Aldrich); 5,6-Dichlorobenzimidazole Riboside (DRB, Pol II transcriptional elongation inhibitor, 100 μM, Sigma Aldrich); Actinomycin D (transcriptional inhibitor, 10 μM, Sigma Aldrich); C646 (CBP-CREB interaction inhibitor, 25 μM, Selleckchem); CAS-92-78-4 (CBP-CREB interaction inhibitor, 4.2 μM, Calbiochem).

### Polysome Assays

For each polysome gradient, two 100-mm dishes of 8–9 d.i.v. cortical neurons were co-transfected with the Firefly Luciferase pGL3 construct and with either Arc UTR or Arc UTR-noI reporter plasmids. After 24 h, cells were treated with BDNF for 4 h or left untreated. Neurons were briefly washed twice in cold PBS and lysed by scraping in lysis buffer [10 mM Tris, pH 7.5; 10 mM MgCl_2_; 100 mM NaCl; 0.5% Triton X-100; 1 mM DTT; 30 U/ml Rnasin (Promega); 30 μg/ml of cycloheximide; 2x “Complete” EDTA-free protease inhibitor complex]. After 15 min at 4°C, the lysate was spun for 10 min at 10,000 × *g*, layered over a 15–50% linear sucrose gradient (15–50% sucrose, 10 mM Tris, pH 7.5; 10 mM MgCl_2_; 100 mM NaCl) and centrifuged in an SW 41 rotor at 37000 rpm for 1.45 h. Fractions (950 μl) were collected with a Bio-Rad Biologic LP gradient fractionator while UV monitored at 254 nm. Equal amounts of spike-in RNA was added to each fraction prior to RNA extraction. RNA was extracted by the addition of an equal volume of 1:1 (vol/vol) phenol/chloroform and precipitated with ethanol and glycogen. The RNA was resuspended in 30 μl of RNase-free H_2_O, treated with DNase I (Promega) and analyzed by real time RT-PCR.

Spike-in RNA was *in vitro* transcribed using a XhoI-linearized pCDNA3.1-GFP plasmid as template. This plasmid was obtained cloning the PCR-amplified GFP coding region in the BamHI- XhoI sites of the pCDNA3.1 polylinker. The resulting transcript corresponds to the GFP coding region. *In vitro* transcription with T7 Polymerase was carried out following manufacturer instructions (Promega).

### Quantitative Real-Time PCR

DNase I-treated total RNA was utilized as template for one-step qRT-PCR using the GoTaq 1-Step RT-qPCR System (SYBR Green) by Promega and following manufacturer instructions. qRT-PCR was performed in a iCycler iQ5 Real-Time Detection system (Bio-Rad, United States). For quantification, the ΔΔCt method was used to calculate relative fold changes normalized against the Firefly mRNA, or the spike-in GFP transcript. Error bars were computed according to the standard error of the mean and the error propagation.

Each sample was analyzed in triplicate and repeated for RNAs collected from at least two independent experiments.

Primer sets utilized are as follows: Renilla reporter (pRLTK-fw; pRLTK-rw); Firefly reporter (Firefly-fw; Firefly-rw); Spike-in transcript (GFP-fw; GFP-rw). To test for the splicing pattern of endogenous Arc mRNA, total RNA extracted from untreated or BDNF-treated neurons was DNase I-treated and subjected to one-step RT-PCR with primers rArcU-4 and rArcU-5. To test for the splicing pattern of our Renilla reporters, total RNA extracted from transfected neurons was DNase I-treated and subjected to one-step RT-PCR with primers pRLTK-ORF and rArcU-6.

## Author Contributions

CG contributed to study concept and design and manuscript preparation; performed most of the experiments, analysis, and interpretation of the data. CP, SR, VDP, CO, and CCa assisted in or contributed to performing luciferase assays, real time PCR, and cloning. MC prepared all the neuronal cultures. CCa, CCo, and AC contributed to study design and drafting of the manuscript. All authors had full access to the manuscript and data for approval.

## Conflict of Interest Statement

The authors declare that the research was conducted in the absence of any commercial or financial relationships that could be construed as a potential conflict of interest.

## Abbreviations

3′ UTR: 3′ untranslated region
5′ UTR: 5′ untranslated region
Act-D: actinomycin-D
AMPAR: α-amino-3-hydroxy-5-methyl-4-isoxazolepropionic acid receptor
Arc: activity-regulated cytoskeleton-associated protein
BDNF: brain-derived neurotrophic factor
cDNA: complementary DNA
CREB: cAMP response element-binding protein
CYC1: cytochrome C1
DG: dentate gyrus
DHPG: (RS)-3,5-dihydroxyphenylglycine
d.i.v.: days *in vitro*
DRB: 5,6-dichloro-1-β-D-ribofuranosylbenzimidazole
DTE: dendritic targeting element
DTT: dithiothreitol
eEF: eukaryotic elongation factor
eIF: eukaryotic initiation factor
EJC: exon junction complex
ELAV/Hu: embryonic lethal abnormal visual system/Hu
ERK: extracellular signal-regulated kinase
FMRP: Fragile X mental retardation protein
fw: forward
GFP: green-fluorescent protein
GPCRs: G protein-coupled receptors
IEG: immediate-early gene
IRES: internal ribosome entry site
JNK: c-Jun N-terminal kinase
LTD: long-term depression
LTP: long-term potentiation
L-LTP: late LTP
mAChR: muscarinic acetylcholine receptor
MAPK: mitogen-activated protein kinase
MEF2: myocyte enhancer factor-2
mGluR: metabotropic glutamate receptor
miRNA: microRNA
Mln51: metastatic lymph node 51
MNK1: MAP-kinase interacting kinase 1
mRNA: messenger RNA
mRNP: messenger ribonucleoprotein
mTOR: mammalian target of rapamycin
mTORC: target of rapamycin (TOR) complex
NGF: nerve growth factor
NMD: nonsense mediated decay
NMDAR: *N*-methyl-D-aspartate
NT-3: neurotrophin 3
ORF: open reading frame
PAS: polyadenylation sequence
PCR: polymerase chain reaction
PI3-K: phosphatidylinositol 3-kinase
PKA: protein kinase A
PKC: protein kinase C
PLC: phospholipase C
Pol II: RNA polymerase II
pRLTK: thymidine kinase promoter Renilla luciferase reporter plasmid
qPCR: quantitative PCR
RT-PCR: reverse transcription PCR
rw: reverse
S6K: p70 S6 kinase
SEM: standard error of the mean
SRF: serum response factor
STRN4: striatin 4
SV40: simian virus 40
TrkA/B/C: tropomyosin-related kinase A/B/C
TTX: tetrodotoxin
Upf1/2/3: regulator of nonsense transcripts 1/2/3.
