# Supplementary material for: Arc 3′ UTR Splicing Leads to Dual and Antagonistic Effects in Fine-Tuning Arc Expression Upon BDNF Signaling
Source: Front Mol Neurosci. 2018 Apr 27;11:145. doi: 10.3389/fnmol.2018.00145 (PMC5934489; doi:10.3389/fnmol.2018.00145)
Supplement: Supplementary file 1 [file Image_1.PDF]

Supplemental Data

Arc 3'UTR splicing leads to dual and antagonistic effects in fine-tuning Arc expression upon BDNF signaling

Chiara Paolantoni, Simona Ricciardi, Veronica De Paolis, Chinenye Okenwa, Caterina Catalanotto, Maria Teresa Ciotti, Antonino Cattaneo, Carlo Cogoni and Corinna Giorgi

Supplemental Figure 1

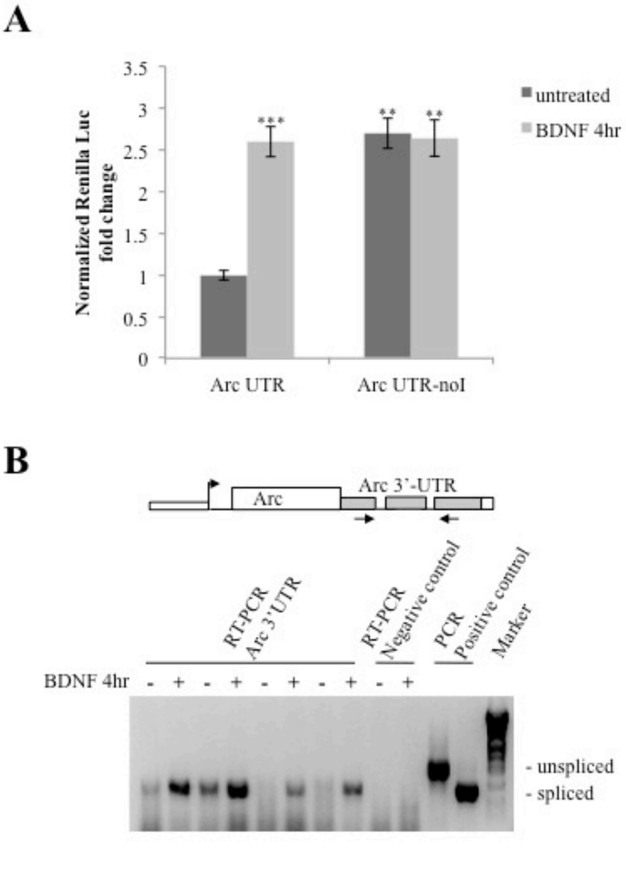

Fig S1

Supplemental Figure 1

(A) Luciferase assay of 13 d.i.v. cortical neurons transfected with Arc UTR or Arc UTR-noI constructs and subjected to a 4 hr chronic treatment with BDNF (100ng/ml) at 16 d.i.v..
The results are expressed as the mean ± standard error (SEM) from five biological replicates each. Student's t-test: *P<0.05, **P<0.01, ***P<0.001, ns: non-significant.
(B) RT-PCR assay to analyze the splicing pattern of endogenous Arc mRNA 3'UTR region prior and upon BDNF treatment. (Upper) Schematic representation of the oligonucleotides utilized in the RT-PCR and in the PCR reactions, spanning both introns of Arc 3'UTR region. (Lower) Non quantitative one-step RT-PCR amplification of RNAs extracted from 4 independent preparations of neurons left untreated or treated with BDNF for 4 hr. As a negative control, RT-PCR reactions were performed omitting the reverse transcriptase enzyme in the reaction mix, and utilizing only Taq Polymerase (RT-PCR Negative control). As a size reference of spliced versus unspliced mRNAs, RT-PCR products were resolved next to PCR amplifications obtained using Arc UTR or Arc UTR-noI plasmid DNA as template (RT-PCR Positive control).
